
JOURNAL INFORMATION
==============================
NLM Title Abbreviation: Behav Neurol
Journal ID: Behav Neurol
NLM Journal ID: 8914585
Journal ID: BN

Behavioural Neurology

ISSN: 0953-4180
EISSN: 1875-8584
Publisher: Wiley

ARTICLE INFORMATION
==============================
PMCID: PMC5452465
PMID: 28615801
DOI: 10.1155/2008/401450
Article ID: 401450
Article version: 1
Subjects: Editorial

A New Impulse for Neuropsychology in Europe: The Federation of the European Societies of Neuropsychology

Print publication date: 2008
Electronic publication date: 2008 Apr 11
Volume: 19
Issue: 1-2
First page: 1
Last page: 1
Received 2008 Mar 31; Accepted 2008 Mar 31
Copyright: Copyright © 2008 Hindawi Publishing Corporation and the authors.
Copyright year: 2008
License: This is an open access article distributed under the Creative Commons Attribution License, which permits unrestricted use, distribution, and reproduction in any medium, provided the original work is properly cited.
License URL: https://creativecommons.org/licenses/by/3.0/

==============================
Behavioural Neurology 19 (2008) 1
IOS Press

Editorial

### A new impulse for neuropsychology in Europe: The Federation of the European Societies of Neuropsychology

Jean-François Démonet MD, PhD, Neurologist,
Directeur de Recherche INSERM
President of the 2nd Meeting of the European Societies of Neuropsychology
Inserm U825, Pôle Neurosciences,
Hôpital Purpan,
F-31059 Toulouse cedex 03, France
Tel.: 33 (0)5 61 77 95 00, 7 76 86:
Fax: 33 (0)5 61 49 95 24;
E-mail: demonet@toulouse.inserm.fr
http:// www-toulouse.inserm.fr

This special issue of Behavioural Neurology includes short papers that originate from a double-step selection among the 350 contributions to the 2nd Meeting of the European Societies of Neuropsychology held in Toulouse in October 2006. This successful conference gathered 500 attendees from many European countries; it allowed the Scientific Committee to select oral and poster presentations of special interest; authors were asked to submit short papers to Behavioural Neurology as Editors, A. Hillis and S. Cappa, kindly offered the Scientific Committee this opportunity to publish the best of our meeting. After peer-reviewing, these 17 short papers represent an interesting panorama of the current trends in Neuropsychology. These trends mainly concern new approaches to visual perception, social cognition, and memory; it is worth of note that several papers address the debatable substrates (Berlingeri et al.), mechanisms (Turriziani et al.) and boundaries of episodic memory as well as its relationships with semantic knowledge (Charnallet et al.), emotion processing and experience (El Sharkawy et al., Tramoni et al.), and autonoetic consciousness (Noulhiane et al., Irish et al.). These stimulating contributions to current hot topics in Neuropsychology are published at the beginning of year 2008 in which will be held next September in Edinburgh, the first formal Meeting of the new Federation of the European Societies of Neuropsychology (http://www.fesn.eu ) that has been joined by virtually all the National Societies of Neuropsychology throughout Europe. The idea of this Federation was launched in Toulouse thanks the decisive drive of Sergio Della Sala. I hope the short papers included herein will constitute a good prelude to the Edinburgh Meeting whose preliminary program already makes one foresee a major event in the multidisciplinary domain of Neuropsychology and Behavioural Neurology.

ISSN 0953-4180/08/$17.00 © 2008 – IOS Press and the authors. All rights reserved
